# Recent advances in animal and human pluripotent stem cell modeling of cardiac laminopathy

**DOI:** 10.1186/s13287-016-0401-5

**Published:** 2016-09-20

**Authors:** Yee-Ki Lee, Yu Jiang, Xin-Ru Ran, Yee-Man Lau, Kwong-Man Ng, Wing-Hon Kevin Lai, Chung-Wah Siu, Hung-Fat Tse

**Affiliations:** 1 Cardiology Division, Department of Medicine, Li Ka Shing Faculty of Medicine, University of Hong Kong, Queen Mary Hospital, Hong Kong, People’s Republic of China; 2 Hong Kong–Guangdong Joint Laboratory on Stem Cell and Regenerative Medicine, University of Hong Kong and Guangzhou Institutes of Biomedicine and Health, Guangzhou, People’s Republic of China; 3 Shenzhen Institutes of Research and Innovation, University of Hong Kong, Hong Kong, SAR China

**Keywords:** Cardiovascular diseases, Lamin A/C, Stem cell model, Transgenic mice model

## Abstract

Laminopathy is a disease closely related to deficiency of the nuclear matrix protein lamin A/C or failure in prelamin A processing, and leads to accumulation of the misfold protein causing progeria. The resultant disrupted lamin function is highly associated with abnormal nuclear architecture, cell senescence, apoptosis, and unstable genome integrity. To date, the effects of loss in nuclear integrity on the susceptible organ, striated muscle, have been commonly associated with muscular dystrophy, dilated cardiac myopathy (DCM), and conduction defeats, but have not been studied intensively. In this review, we aim to summarize recent breakthroughs in an in vivo laminopathy model and in vitro study using patient-specific human induced pluripotent stem cells (iPSCs) that reproduce the pathophysiological phenotype for further drug screening. We describe several in-vivo transgenic mouse models to elucidate the effects of *Lmna* H222P, N195K mutations, and *LMNA* knockout on cardiac function, in terms of hemodynamic and electrical signal propagation; certain strategies targeted on stress-related MAPK are mentioned. We will also discuss human iPSC cardiomyocytes serving as a platform to reveal the underlying mechanisms, such as the altered mechanical sensation in electrical coupling of the heart conduction system and ion channel alternation in relation to altered nuclear architecture, and furthermore to enable screening of drugs that can attenuate this cardiac premature aging phenotype by inhibition of prelamin misfolding and oxidative stress, and also enhancement of autophagy protein clearance and cardiac-protective microRNA.

## Background

The *LMNA* gene locates in the long branch of chromosome 1, producing two main isoforms by alternative splicing (i.e., lamin A and C). These isoforms are the intermediate filaments and constitute the major components of the nuclear lamina [1]. Lamin A and C are present in most somatic cells that have a multimeric fibrous structure surrounding the nucleus and provide support to the nuclear membrane proteins. In recent years, the role of lamin A/C has been investigated, for example, in the maintenance of chromatin organization during cell division, signal transduction, differentiation maintenance, repair, and anchoring of other lamin-binding proteins, such as emerins, desmin, and nesprin. Mutations in *LMNA* have been shown to cause a wide range of human diseases, collectively referred to as “laminopathies” [2–4]. These include Hutchinson Gilford progeria syndrome (HGPS, premature aging syndrome) caused by a truncated splicing mutation of the *LMNA* gene, resulting in the generation of progerin, muscular dystrophy, and familial dilated cardiomyopathy (DCM). The mutations may also affect muscle, fat, bone, nerve, and skin tissues and lead to inherited neuromuscular disease with multiple phenotypic expressions such as Emery–Dreifuss muscular dystrophy (EDMD), limb girdle muscular dystrophy 1B (LGMD1B), Dunnigan-type familial partial lipodystrophy, a recessive axonal form of Charcot–Marie–Tooth neuropathy, and mandibuloacral dysplasia. However, there is a lack of understanding about the underlying mechanisms concerning lamin insufficiency or misfolding of such protein in cardiac disease progression. Current existing platforms for cardiolaminopathy modeling rely on transgenic mice to determine gene dose effects of the heterogeneous and homogeneous mutation system, the animal replicated clinical phenotypes with muscle dystrophy, premature DCM syndromes, as well as atrioventricular (AV) block. Although rodent systems allow studies of whole heart function, the cardiac physiological makeup is deviated from the human condition. Recent breakthroughs in generation of human induced pluripotent stem cell (iPSC) technologies allow access to patient-specific materials (e.g., heart, gut, neurons, and liver cells) that recapitulate the disease phenotype in a culture system. Recently, scientists have relied on such a system for electrophysiological study at a single cell level, as a platform to determine deterioration of nuclear architecture due to premature cell senescence, and also to determine energy synthesis dynamics. More importantly, the human cardiac cell would allow pilot drug-screening studies on targeting oxidative stress signaling in cardiac laminopathy, clearance of misfolded lamin proteins, delay in the rate of producing toxic farnesylated lamin, arising from mutation at cleavage sites of prelamin A/C protein, the blockade of stress-related MEK1–Erk1/2, JNK, and p38-mediated MAPK pathways, or even the cardiac protective microRNA (miR) that reduces prelamin A accumulation. More recently, the breakthroughs in gene editing technologies allow allogeneic cell therapies or generation of isogenic control. The use of iPSC derivatives could be used as a critical and powerful tool for standardized and comparative pharmacological studies.

## Clinical observations in cardiac laminopathy

Various genetic causes have been identified that play a vital role in the formation of DCM, although in most cases the underlying mechanism remains unknown. More than 60 genes have been identified, including the lamin A/C gene (*LMNA*), that cause monogenic DCM [5]. *LMNA-*related DCM is characterized by early onset of atrial fibrillation, conduction system disease, and subsequent progression to sudden cardiac death and premature heart failure [6–9]. To date, 20 % of gene mutations associated with DCM are believed to be linked to Titin (*TTN*). *LMNA* mutations are the second most common cause of familial DCM, responsible for 5–10 % of overall familial DCM and up to 30–45 % of families with DCM and conduction system disease [10, 11]. Although the age at presentation of *LMNA-*related DCM ranges from the first to sixth decade of life, the laminopathy-mediated cardiac defeats are always progressive and almost all patients become symptomatic after age 60 [7, 8, 12]. Furthermore, *LMNA-*related DCM, especially that associated with conductive system diseases, has a more malignant clinical course than other familial DCM because of the high rates of progressive heart failure and sudden cardiac death due to ventricular tachyarrhythmias, and the ultimate treatment would rely on heart transplantation [12–15]. Despite our increasing awareness of the importance of *LMNA-*related DCM, the mechanisms of the disease as well as therapeutic strategies to prevent its onset and progression remain unclear. Early clinical manifestations are often apparent in the conduction system and specifically lead to sick sinus syndrome, and AV block or bundle branch block with approximately 28 % of affected patients requiring permanent pacemaker implantation [16, 17]. A meta-analysis of 299 patients with an *LMNA* gene mutation suggested that cardiomyopathy due to *LMNA* mutations indicates a high probability of sudden death [17]. The analysis revealed that 92 % of patients over the age of 30 years suffered cardiac arrhythmias, 64 % after age 50 years suffered heart failure, and both the cardiac and neuromuscular phenotype was reported in 46 % of cases of sudden death. A pacemaker was implanted in 28 % of lamin A/C gene mutation carriers, although this did not alter the rate of sudden death.

More recently, Andre et al.’s study described a *LMNA* T655fsX49 mutation that led to lipodystrophic laminopathy. In fact, the mutation was associated with failure in processing of prelamin A which resulted in accumulation of nonfarnesylated mutated prelamin A. It was further shown that there is a relationship between mutated prelamin A accumulation and the severity of the phenotypes in homozygous familial partial lipodystrophy type 2 patients who harbor the *LMNA* T655fsX49 mutation [18] (Table 1).

**Table 1:** Phenotype of the mutated *LMNA* mouse model and the human iPSC model

Model	*LMNA* mutation	Phenotype	Reference
Animal	Knockout	Retarded growth rate and early death	[26]
	Conditional knockout	Hindered growth; postnatal cardiomyocyte hypertrophy, skeletal muscle dystrophy	[28, 29]
	H222P	Cardiac conduction defeats, chamber dilation and enhanced incidence of fibrosis; muscular dystrophy	[20, 24, 52, 53]
	N195K	DCM and conduction system disease; irregular heart rhythm	[25]
Human	HGPS	Epigenetic alternation associated with premature aging; vascular aging; premature osteogenesis	[42, 44, 45, 48]
	T655fsX49	Lipodystrophy type 2; muscle hypertrophy; Atrial fibrillation (AF); cardiac conduction disease with first-degree AV block and homozygous patients showed frequent secondary-degree AV block; DCM; ventricular arrhythmia	[18]
	R225X	Patients showed early onset of AF, secondary AV block and DCM; retarded human iPSC-derived cell proliferation, premature cell senescence; viability of CMCs susceptible to stress condition (e.g. electrical field stimulation)	[6, 52, 54–56]

*AV* atrioventricular, *CMC* cardiomyocyte, *DCM* dilated cardiomyopathy, *HGPS* Hutchinson Gilford progeria syndrome, *iPSC* induced pluripotent stem cell

## Animal models of cardiac laminopathy

To provide initial insight into the pathophysiology of *LMNA*-mediated DCM and muscular dystrophy, several transgenic animal models of *LMNA* mutations have been generated [19–21]. Either *LMNA* mutation knockin (KI) (dominant negative) [20] or *LMNA* knockout (KO) (haploinsufficiency) transgene presented apart from DCM phenotypes [19, 21], but also variable phenotypes of the conduction system disease (Table 1). In 2003, the first KO mouse model of A-type lamin (Lmna^−/−^) was established by Sullivan and colleagues [22, 23]. In early age, these homozygous KO mice rapidly displayed a retarded growth rate, which agreed with the phenotypes presented in HGPS. Subsequently, all homozygous mice died by the fourth week after birth. Apart from the suppressed level of *lmna*, Bonne and colleagues introduced an H222P mutation in *LMNA* in a mouse model, which displayed typical cardiac conduction defects, chamber dilation, and increased fibrosis but showed a lack of hypertrophy [24]. In fact, the Lmna-H222P mice also showed signs of muscular dystrophy and underwent premature death at 4–9 months for males and at 7–13 months for females. With the confirmation of phenotypes resembling a patient’s condition, this model was employed as a platform for drug screening of which drugs act on stress-related pathways. Apart from the H222P mutation, the group of Leslie and Serguei observed the phenotype of homozygous KI-*Lmna* N195K mice [25] that recapitulated the phenotype of DCM and conduction system disease. The homozygous N195K mice showed early signs of DCM, increased interstitial fibrosis, irregular heart rhythm, and conduction defects, with a high mortality rate at 6–8 months. The mutant mice were observed to have sarcomeric and desmin disorganization, mislocalization of connexin 43, and decreased expression of connexin 40. Although the mutation suppressed lamin A/C expression, which was properly localized at the nuclear envelope, the emerin connecting intermediate filament with lamin A/C is partially mislocalized to the cytoplasm [26, 27].

**Fig. 1:**
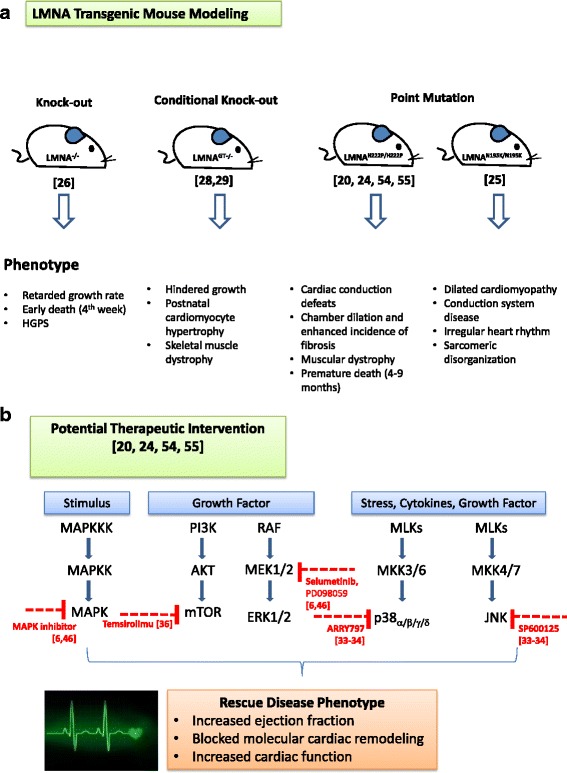
**a** Schematic diagram of existing laminopathy animal modeling and the phenotypes. **b** Development of pharmacological treatment on targeted pathways affected by laminopathy. *HGPS* Hutchinson Gilford progeria syndrome, *MAPK* mitogen-activated protein kinase, *MEK1* MAPK–extracellular signal-regulated kinase-1

In 2011, Kubben et al. [28] developed a novel *LMNA* null mouse (*LMNA* GT^–/–^) by inserting a promoter in intron 2 of *LMNA*, resulting in a *LMNA*-β-geo fusion allele. This model combined the *LMNA* gene KO with LMNA-driven reporter, and thus enabled in-vivo study of the effect of conditional lamin A/C ablations during early postnatal development. In these KO mice, hindered growth, postnatal cardiomyocyte hypertrophy, skeletal muscle dystrophy, and metabolic defects were observed in the first 2 weeks after birth. Premature fatal events were commonly observed in mice before weaning. Similar results were later observed in a conditional *LMNA* KO mouse model created by Kim and Zheng [29], with the introduction of *LoxP* sites flanking the *LMNA* exon 2, which were further crossed with CMV-Cre mice to create a conditional KO driven by *LMNA* expression. The generated *LMNA*^–/–^ mice exhibited growth delay from the first 12 days and died between postnatal days 16 and 18. It was also suggested that loss of function in muscle was due to the decreased skeletal myofibril size, similar to observations in Lmna GT^−/−^ mice [28]. Overall, lamin A/C loss may strongly affect the transcription of genes related to muscle differentiation and thus account for the delayed muscle maturation observed in various Lmna KO mouse models (Fig. 1a﻿).

## Development of a potential therapeutic intervention using a transgenic animal model

Despite our increasing awareness of the importance of *LMNA-*related DCM, the mechanism of the disease as well as therapeutic strategies to prevent its onset and progression remain unclear. DCM with Lmna mutation is always very aggressive. Common clinical manifestations are related to development of heart failure and sudden cardiac death, for which ultimate treatment/prevention relies on cardiac transplantation [30]. Although conventional pharmacotherapy relies on angiotensin-converting enzyme inhibitors (ACEI), there is no specific treatment for the progressive loss of contractility in *LMNA*-related cardiomyopathy. A mechanistic understanding of the physiopathological basis of such disease is necessary to develop more specific and efficacious therapeutic strategies.

In recent decades, the incidence of fatal tachyarrhythmia has been greatly reduced by prophylactic implantation of a cardioverter defibrillator [13]. Anselme et al*.* [31] reported that the high incidence of life-threatening tachyarrhythmia in patients with *LMNA* mutation necessitated implantation of a cardioverter defibrillator instead of a pacemaker. In 2007, in order to investigate the pathogenesis of *LMNA* cardiomyopathy, Muchir et al. implemented a genome-wide transcriptome analysis of hearts isolated from *Lmna* H222P mice. Significant differences were noted in the expression of gene encoding proteins in stress-activated MAPK and mTOR signaling pathways in the mutated mice. Their work clearly verified an abnormal increase in both MAPK and mTOR activity in heart tissue from *Lmna* H222P mice [20]. These results indicated that MAPK and mTOR inhibition may offer an alternative therapeutic option to delay the onset of heart failure in LMNA-related cardiomyopathy. To determine treatment for mutated *LMNA-*induced cardiac disorders, Muchir et al. also treated *Lmna* H222P mice with daily intraperitoneal injections of the MEK1/2 inhibitor (Selumetinib). Selumetinib treatment resulted in left ventricular (LV) end-systolic dilatation, increased ejection fraction, and blocked molecular cardiac remodeling (i.e., blocked increased cardiac natriuretic factor transcripts and halted the induction of elements of the “fetal gene program”), with consequent improved cardiac function compared with placebo-treated mice. Since cardiac fibrosis is a common manifestation in end-stage DCM, and particularly in *LMNA* cardiomyopathy, cardiac fibrosis was also examined in this experiment. The Selumetinib-treated group had a lower degree of cardiac fibrosis than the placebo group. The same research group also revealed that germline deletion of ERK1 in the same mutant mice resulted in enhanced heart function at an early age (16 weeks old) [32], although the improvement could not be sustained beyond 20 weeks of age. ERK2 has also been strongly activated by more than two-fold in *Lmna* H222P mice. After cardiac ERK2 activity was blocked with Selumetinib, the ejection fraction at 20 weeks was significantly enhanced, implying that the increased ERK2 activity compensated for the ERK1 ablation and resulted in deteriorated heart function in the *Lmna* H222P mice that lacked ERK1 activity. They also found that inhibiting JNK (SP600125) [33, 34] or p38 (ARRY797) exerted beneficial effects on LV dysfunction in the mice. In addition to the enhanced ERK1/2 signaling, activities of the other stress response MAPKs, JNK and p38, were also enhanced at an early stage of disease in *Lmna* H222P mice hearts [20, 35]. Therefore, p38 and JNK activity increased in *Lmna* H222P/Erk1 null mice compared with control *LMNA*-WT/Erk1 null mice when LV function started to change. We have previously reported the benefits of inhibiting JNK (SP600125) [33, 34] or p38 (ARRY797) in LV dysfunction in LmnaH222P mice. In future experiments, a combination of inhibitors of p38 and JNK in LmnaH222P/Erk1null mice may be used to identify their effect on heart function and may help clarify the individual or overlapping functions of these diverse signaling pathways in heart pathology affected by the *LMNA* mutation.

After the experiment with MAPK inhibitors, Muchir’s team treated the *Lmna* H222P mice for 2 weeks with a mTOR inhibitor, Temsirolimu, for clearance of waste protein generated by autophagy [36]. Similar to the results of Selumetinib treatment, improved heart function of the treated mice presented with enhanced LV end-systolic dilation and ejection fraction and attenuated cardiac remodeling (Fig. 1b).

## Human induced pluripotent stem cell modeling of laminopathy and drug screening

The high mortality of these *LMNA* knockout mice restricted the possibility of chronic whole animal study. In addition, differences in cardiac electrophysiological behavior between humans and rodents may hinder the feasibility of translating pathophysiological discoveries into clinical practice. The mechanisms by which different *LMNA* mutations cause AV block or DCM remain uncertain. An in-vitro platform of human cardiomyocytes derived from patients with different *LMNA* mutations would be extremely useful for understanding disease mechanisms under stress conditions such as electrical field stimulation and mechanical stretch, as well as a hypoxic environment, and hence developing patient-specific therapies.

**Fig. 2:**
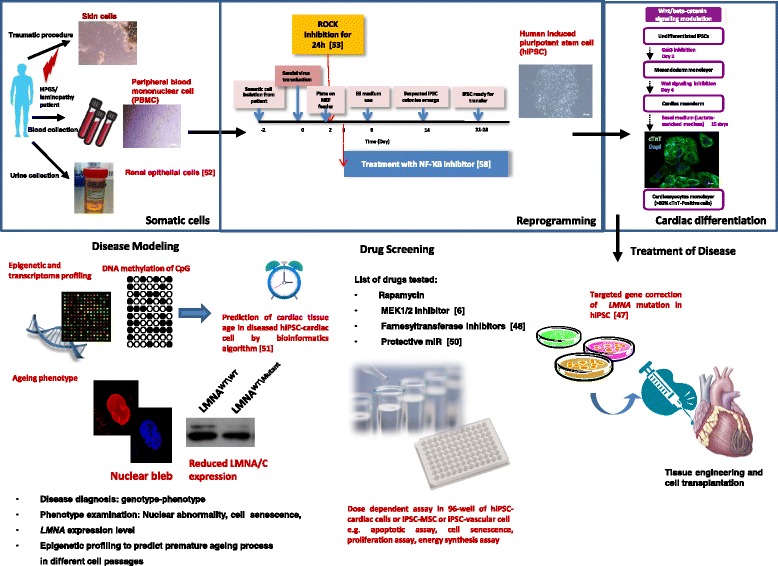
Schematic summary of existing cardiac laminopathy human iPSC modeling and future studies to understand the disease mechanism, drug screening, and interventions. *HGPS* Hutchinson Gilford progeria syndrome, *miR* microRNA, MLK Mixed-lineage kinases. [57, 58]

The recent breakthrough of human iPSCs generated from adult somatic tissues [37, 38] provides a unique opportunity to produce patient-specific cardiomyocytes for disease modeling and drug screening [39–41] (Table 1 and Fig. 2). Since iPSCs are genetically identical to the host bearing cardiac defeats, the iPSC-derived cardiomyoctes provide an attractive experimental platform to recapitulate cellular phenotypes of familial heart diseases such as arrhythmias and cardiomyopathies. This will provide new insights into disease-modifying mechanisms and enable the specific design of personalized therapeutic strategies.

In 2011, Liu et al. [42] began to use human iPSCs for HGPS modeling. HGPS is caused by a single point mutation in the lamin A (*LMNA*) gene, resulting in the generation of progerin, a truncated splicing mutant of lamin A. The level of progerin accumulates with ages and leads to various ageing-associated nuclear defects including disorganization of the nuclear lamina and loss of heterochromatin. The reversible suppression of progerin expression by reprogramming was resumed upon differentiation with ageing-associated phenotypic consequences. The HGPS-iPSCs derived from skin fibroblasts showed an absence of progerin and more importantly lacked the nuclear envelope and epigenetic alterations normally associated with premature ageing. Nevertheless, the appearance of premature senescence phenotypes in HGPS-iPSC-derived smooth muscle cells (SMCs) was associated with vascular ageing. Additionally, they identified a DNA-dependent protein kinase catalytic subunit (DNAPKcs, also known as PRKDC) as a downstream target of progerin. The absence of nuclear DNAPK holoenzyme correlated with premature as well as physiological ageing. Others have reported the use of a human iPSC platform to model the disease phenotypes of HGPS in mesenchymal lineages and SMCs [42, 43]. Ho et al. as well as Liu et al. generated progeria iPSCs from skin fibroblasts of a patient bearing a mutation in *LMNA* [42, 44]. They proved that the human iPSC-derived fibroblasts are able to recapitulate the disease phenotype with prominent nuclear blebbing, are capable of cell senescence, and are susceptible to external stimulation (e.g., electrical field stimulation as the donor cells). Liu et al. showed that premature vascular ageing was probably due to accumulation of progerin in SMCs. Later, Blondel et al. in 2014 further investigated the translational aspect using iPSCs to reveal functional differences between drugs currently investigated in patients with HGPS. They trialed a farnesyltransferase inhibitor in combination with a statin (zoledronate and pravastatin), and the macrolide antibiotic rapamycin. This study revealed that a systematic cytostatic effect was observed in the treatment group with the farnesyltransferase inhibitor alone [45]. The investigators provide new insights into drug efficacy in functional improvement of prelamin A farnesylation that generates cytotoxic progerin, nuclear architecture, improvement in cell proliferation, as well as energy metabolism; in other words, ATP synthesis. This finding further proved iPSCs to be powerful tools for standardized and comparative pharmacological studies.

In 2012, our group subsequently generated another human iPSC platform from a patient bearing a premature termination codon in the *LMNA* gene, R225X. Although no clear nuclear phenotype was observed in iPSCs from the DCM patient with the *LMNA* mutation, several cellular phenotypes were observed in the human iPSC-derived cardiomyocytes, including nuclear morphology abnormality (blebbing), slow proliferation, improved cellular senescence, and increased incidence of apoptosis under electrical stimulation. Under field electrical stimulation to mimic the native cardiac environment, the percentage of *LMNA*‐mutated iPSC cardiomyocytes that exhibited nuclear senescence and cellular apoptosis markedly increased. shRNA knockdown of *LMNA*, resembling the halploinsufficiency situation of the R225X mutant, replicated those phenotypes of the mutated *LMNA* field electrical stress. We also demonstrated the central role of the MAPK–extracellular signal-regulated kinase-1 (MEK1) pathway in governing susceptibility to cardiac cell stress-response. Blockage of the extracellular signal-regulated kinase (ERK) pathway by MEK1 inhibitors attenuated the electrical stimulation-induced proapoptotic phenotypes of DCM iPSC cardiomyocytes [6]. ERK1/2 are activated directly by the upstream MEK1/2, which are dual-specificity protein kinases. Activated ERK1/2 kinases phosphorylate and activate a variety of substrates, which can be transcription factors, protein kinases and phosphatases, cytoskeletal and scaffold proteins, receptors and signaling molecules, and apoptosis-related proteins. Numerous MEK1/2 inhibitors have progressed into clinical trials since the identification of the first MEK inhibitor, PD098059 [46]. Most of these MEK1/2 inhibitors are ATP noncompetitive and bind to a unique allosteric site adjacent to the ATP site. Apart from pharmacological treatment of *LMNA* mutation-related disease, there were new breakthroughs in gene editing technologies for correction of laminopathy-associated LMNA mutations in patient-specific iPSCs. However, Liu et al. [42] discovered that the *LMNA* gene was transcriptionally inactive and would impede targeted gene editing. They further explored using helper-dependent adenoviral vectors (HDAdVs) as a robust and highly efficient vehicle for the delivery of gene editing tools. In comparison with the conventional piggybac method, the advantage of this system is the inclusion of a negative selection step by ganciclovir (GNAC) resistance to eliminate random insertion of clones that contain the HSV*tk* cassette. The resultant corrected HPGS iPSCs were essentially proved to be genetically identical to fibroblasts as well as epigenetically similar to the uncorrected clones. Such a new method would enhance the reliability of gene correction as a therapeutic tool to rescue the disease phenotype for cell therapies or to generate a patient-matched control for disease modeling and further the dissected disease causal target for drug discovery [47].

In fact, somatic reprogramming of the progeria patient-specific cell to a human iPSC is not an easy task with the considerable drawback of low efficiency of stem cell clone formation. The stress of premature aged cells was basically due to oxidative stress-related NF-kB activation, which blocks the generation of iPSCs and MSC differentiation. Soria-Valles et al. discovered that NF-kB repression occurred during reprogramming towards a pluripotent state. In contrast, the hyperactivation of NF-kB impaired the process though DOT1L, a histone H3 methyltransferase, which reinforced the senescence signals [48]. In the light of such observations, the authors demonstrated attenuating the NF-kB signal via direct or upstream DOT1L inhibition before somatic reprogramming, which also extended the lifespan and ameliorated the accelerated ageing phenotype in the animal model. Chronic treatment of NF-kB inhibition, an anti-inflammatory compound, may produce side effects. Besides, DOT1L inhibitors have recently been tested for the treatment of hematological malignancies, which suggests a better solution for age-associated diseases [49].

Apart from epigenetic profiling, the tissue-specific expression profile of miR may provide clues for laminopathy therapies. miR-9 was specifically expressed in neuronal cells derived from HGPS patients, which exerted a protective role of the miR specifically to preserve cognitive function [50]. The miR-9 acting 3′-untranslated region (UTR) of lamin A suppresses its expression level, thus reducing accumulation of prelamin A, which generates progerin. The direct role of miR-9 on lamin A gene expression was further confirmed by anti-miR-9 treatment (loss of function) or transfection with pre-miR-9 (gain of function) in the HGPS iPSC-MSC. Future studies on cardiac-specific laminopathy intervention could be focus on inhibiting miR-9 or other cardiac-specific miR targeting on the 3′-UTR of *LMNA*.

## Conclusions and further studies

Different types of mutations in *LMNA* present varying severity of cardiac laminopathy phenotypes, such as alternation in splice variant maturation causing progerin accumulation and haploid insufficiency. The mutations could cause familial cardiomyopathy, early onset of AV block, and lethal ventricular tachycardia. The findings of translational implication facilitate screening of *LMNA* mutation which might be beneficial for risk stratification and clinical management of this type of familial cardiomyopathy or arrhythmia. Further studies concerning the effects of different lengths of truncated lamin proteins, such as location in proximity to the prelamin A cleavage site, need to be revealed. The overexpression of the unstable form of truncated proteins would generate an artificial system to extrapolate their prominent role in disease progression or the severe disease phenotype was only based on a reduced level of full-length Lmna. In an animal model, cardiac laminopathy has been found to be closely related to heart block, atrial fibrillation, and DCM. The transgenic animal would allow cardiac hemodynamic functional study and pharmacological testing. However, the direct role of a specific mutation in presentation of different forms of arrhythmia remains unknown. Further in-depth investigation in the human cell environment concerning the role of lamin in ion channel trafficking and the contribution of tight junction protein (e.g., CX40 and CX43) in the conduction system to the cell conductance would be necessary. Up to now, it is clear that cardiac defeat mediated by *LMNA* mutation could be ameliorated by manipulation of the Akt/mTOR pathway by facilitating clearance of accumulated mutant protein through the process of autophagy and MEK1-mediated Erk1/2 by inhibition of apoptotic stress responses. As a consequence, further studies would also rely on a human iPSC model to investigate more clinical relevant outcomes. It would be interesting to explore cardiac-specific presentation of a laminopathy phenotype based on mechanical sensitivity of nuclear lamins coupled to membrane surface receptors.

In the new era of advances in epigenetic studies, we could use a bioinformatics algorithm as a mathematical model to predict the age of human tissues based on profiles of cytosine-5 methylation within CpG dinucleotides, also known as DNA methylation (DNAm). The use of such an epigenetic clock based on 353 CpG sites could be validated in multiple tissues to predict the research gap in premature aging studies. It is well known that age-related DNA hypomethylation has long been observed in rodents [51]. The authors pointed out an important issue for iPSC modeling of premature aging disease since the stem cells tend to have their DNAm age reset to zero compared with the corresponding somatic cells. They suggested performing multiple cell passaging to accelerate the DNAm age that resembles the actual situation. Indeed, the DNAm profile is not only age specific, but also tissue type specific; one should calibrate to a specific target type profile before interpretation. Given the high heritability of age acceleration in young subjects, iPSCs could be a powerful model to study ageing dynamics in terms of genomic stability to maintain DNAm in cardiac laminopathy from embryonic to adult stages. In the future, DNAm age may become a powerful surrogate marker for evaluating rejuvenation therapies for drug screening in progeria or laminopathy diseases.

## Abbreviations

ACEI: Angiotensin-converting enzyme inhibitors
AF: Atrial fibrillation
DCM: Dilated cardiomyopathy
DNAm: DNA methylation
DNAPKcs: DNA-dependent protein kinase catalytic subunit
EDMD: Emery–Dreifuss muscular dystrophy
ERK: Extracellular signal-regulated kinase
GNAC: Ganciclovir
HDAdVs: Helper-dependent adenoviral vectors
HGPS: Hutchinson Gilford progeria syndrome
iPSC: Induced pluripotent stem cell
KI: Knockin
KO: Knockout
LGMD1B: Limb girdle muscular dystrophy 1B
LV: Left ventricular
MAPK: Mitogen-activated protein kinase
MEK1: MAPK–extracellular signal-regulated kinase-1
miR: MicroRNA
PRKDC: Protein kinase catalytic subunit
SMC: Smooth muscle cell
TTN: Titin
UTR: Untranslated region
